# Other News

**Published:** 1922-04

**Authors:** 


### Other News.

Free advice on whether or not to marry is shortly to be available in Vienna for people who wish to observe the principles of eugenics.

The Wigmore Hotel is being converted into a nursing home with 50 bedrooms, each with its own bathroom, and two operating theatres. Several medical men will be upon the board.

A series of five nursing homes for voluntary mental cases has been established in or near Edinburgh, where fifty boarders in all can be accommodated without certification. The scheme has been quietly organised because the demand upon the homes is very great.

The Welsh National School of Medicine has established the first diploma for tuberculous diseases in this country. The diploma is in connection with the Tuberculosis Chair recently founded at the School of Medicine, Cardiff, by Major David Davies, M.P., and now occupied by Professor Lyle Cummins.

The Royal Air Force Nursing Service now offers opportunities for service abroad, and nurses wishing to join this service should, therefore, be willing, if required, to take their turn at service overseas, generally after a term of Home service. Particulars may be obtained on application to the Matron-in-Chief, Air Ministry, Kingsway, London, W.C.2.

The officers of the third International Congress of the History of Medicine are: President of honour, Sir Norman Moore, Bt., M.D.; president, Dr. Charles Singer; treasurer, Mr. W. G. Spencer, O.B.E., M.S.; general secretary, Dr. J. D. Rolleston; vice-presidents, Sir D'Arcy Power, K.B.E., F.R.C.S., Dr. Tricot-Royer of Antwerp, president of the first International Congress, and Drs. Jeanselme and Menetrier, of Paris, presidents of the second International Congress.

The Lord Mayor of London and Lady Mayoress will attend the Congress of the Royal Sanitary Institute at Bournemouth on July 24-29, and will act as Hon. Presidents of the Conference of Representatives of Sanitary Authorities and Section C., Personal and Domestic Hygiene. Official delegates will be present from New South Wales, Victoria, Tasmania, Canada, South Africa, Hong Kong, Shanghai, Denmark, Singapore and Ceylon.

An Army Council Instruction directs that the attention of all ranks should be drawn to the necessity for taking the greatest care of their teeth in order to avoid not only needless suffering but inefficiency. Dental officers will give instruction to soldiers in oral hygiene, both by lectures and demonstrations. An annual dental inspection will be held to ensure that all treatment necessary to maintain dental efficiency is carried out. Dentures will be supplied only to soldiers serving on normal engagements, and only where the approval of the inspecting dental officer has been obtained.

The U.S. Public Health Service has felt it necessary to prevent the too optimistic and extravagant claims recently appearing in the newspapers in regard to the curative effects of chaulmoogra oil derivatives on leprosy. While the use of the oil and of its derivatives has resulted in a considerable number of apparent cures, it is as yet too soon to tell whether these will be permanent. The ethyl esters of chaulmoogra oil, the use of which has largely supplanted the oil itself, constitute a most valuable agent in the treatment of leprosy. In treating young persons and those in the early stages of the disease, the improvement has been rapid and striking; in older persons and older cases it is less so. Of the cases on parole from the leprosy stations in the Hawaiian Islands so far about eight per cent. have relapsed and returned for treatment. This was to be expected, and on the whole the results have been so favourable as to make treatment of the disease hopeful, but only time can tell.

### MEMORANDA FOR APRIL.

On April 6, at 8.15, a discussion on "Occupational Education" will be held under the auspices of the Civic Education League at Leplay House, 65, Belgrave Road, Westminster, S.W.1. The opening paper will be read by Miss M. M. Barker, B.Sc.

Dr. Andrew Balfour, C.B., C.M.G., M.D., will give a lecture on "The Outlook in Tropical Hygiene" on Wednesday, April 26, at 5.30 p.m., at the Royal Sanitary Institute, 90, Buckingham Palace Road, S.W.1. It will be remembered that the Council of the Institute has established an examination in tropical hygiene, in sequence with the present standard examination for sanitary inspectors. Examinations will be held in London this year on April 21-22, July 14-15, and December 8-9. Particulars can be obtained from the Registrar of the Institute.

By permission of the Lord Mayor and the Lady Mayoress, a concert will be held in the Egyptian Hall at the Mansion House, on Friday, April 21, at 3 p.m., in aid of the Prince of Wales's General Hospital. Lady Milsom Rees has kindly undertaken the organisation. Miss Lilian Braithwaite, Miss Irene Vanbrugh, Miss Carrie Tubb, Mr. Ben Davies, Mr. Nelson Keys, Mr. Dawson Milward, and other well-known artists have promised to give their services. Tickets, one guinea and 10s. 6d., may be obtained from the Director, the Prince of Wales's General Hospital, N.15; Lady Milsom Rees, 18, Upper Wimpole Street, W.1; Mrs. F. Brown, 36, Amhurst Park, Stamford Hill, N.16; Mrs. Carson, 111, Harley Street, W.1; or Mrs. Gillespie, 24, Weymouth Street, W.1.

### THE COLLEGE OF NURSING.

This year the annual meeting and conference of the College of Nursing is to take place in London during June. It is hoped that there will be a record gathering at the first reception, to be held at the social headquarters of the College members, the Cowdray Club, shortly to be opened, at 20, Cavendish Square, W.1. The fine mansion is still in the hands of the decorators as we write, but we hear that it "will easily put into the shade any other women's club in London."

The College Library of Nursing, which owes its origin to a grant of £500 made by the Carnegie United Kingdom Trust, is now open to all nurses and students, whether members of the College or otherwise. Intending borrowers should obtain application forms from the Librarian. Gifts of suitable books are solicited for the building up of the Library.

Five scholarships are open for competition among trained nurses desiring to qualify as sister-tutors. Successful candidates are entitled to a course of one year's training at King's College for Women, beginning in October next. Candidates must be members of the College at the time when their application forms are received. These forms are issued by the Secretary, 7, Henrietta Street, Cavendish Square, London, W.1, and must be completed and handed in on or before June 14.

We are requested to state that nomination papers for the election to the Council of the College can now be obtained from the Secretary, but are not being sent to members as in previous years. They must be received by the Secretary not later than April 1.

The Treasure Cot Co. has now removed to larger and more accessible premises at 103, Oxford Street, London, W.1, with extensive showrooms on the first and second floors, where their well-known specialities are displayed.

Hazelwood House, Dumbreck, has been privately handed to the Committee of the Glasgow and West of Scotland Home for Aged and Retired Nurses, and, when complete, will accommodate 25 in separate rooms. The Home is being opened in connection with the King Edward VII. Memorial Fund for Nurses. There is already a similar home in Edinburgh.

